# The First Detection and Genetic Characterization of Four Different Honeybee Viruses in Wild Bumblebees from Croatia

**DOI:** 10.3390/pathogens10070808

**Published:** 2021-06-25

**Authors:** Ivana Tlak Gajger, Laura Šimenc, Ivan Toplak

**Affiliations:** 1 Department for Biology and Pathology of Fish and Bees, Faculty of Veterinary Medicine, University of Zagreb, 10000 Zagreb, Croatia; 2 Virology Unit, Institute of Microbiology and Parasitology, Veterinary Faculty, University of Ljubljana, Gerbičeva 60, 1000 Ljubljana, Slovenia; laura.simenc@vf.uni-lj.si (L.Š.); Ivan.Toplak@vf.uni-lj.si (I.T.)

**Keywords:** *Bombus* spp., honeybee viruses, deformed wing virus, black queen cell virus, acute bee paralysis virus, chronic bee paralysis virus, genetic characterization, sequencing, transmission routes

## Abstract

To determine the presence and the prevalence of four different honeybee viruses (acute bee paralysis virus—ABPV, black queen cell virus—BQCV, chronic bee paralysis virus—CBPV, deformed wing virus—DWV) in wild bumblebees, pooled randomly selected bumblebee samples were collected from twenty-seven different locations in the territory of Croatia. All samples were prepared and examined using the RT-PCR methods for quantification of mentioned honeybee viruses. Determined prevalence (%) of identified positive viruses were in the following decreasing order: BQCV > DWV > ABPV, CBPV. Additionally, direct sequencing of samples positive for BQCV (*n* = 24) and DWV (*n* = 2) was performed, as well as a test of molecular phylogeny comparison with those available in GenBank. Selected positive field viruses’ strains showed 95.7 to 100% (BQCV) and 98.09% (DWV) nucleotide identity with previously detected and deposited honeybee virus strains in the geographic areas in Croatia and neighboring Slovenia. In this article, the first detection of four honeybee viruses with genetic characterization of high diversity strains circulating in wild bumblebees in Croatia is presented.

## 1. Introduction

Requirements for food production and agricultural intensification are resulting in a growing demand for insect pollination services [1]. Bumblebees (*Bombus* spp.) are vital and important insect pollinators to both the agricultural crops and wild plants in natural ecosystems, worldwide [2]. Their commercial rearing has boosted the economic importance of this insect in crop pollination [3]. Free-living bumblebee colonies are integral pollinators within native plant communities throughout temperate ecosystems. Adult bumblebees’ robust size, long tongues, and buzz-pollination behavior result in their great pollination effectiveness. Therefore, they are indispensable, particularly for some plant species.

The decline in the number of insects pollinators is determined by various factors, such as climatic niche changes combined with reductions in natural food availability, the lack of the nesting materials, landscape alteration, agricultural intensification, and the spread of pathogens [4,5]. Reports of pollinator numbers declining due to the increased mortality in recent years are alarming on a global level [5,6,7].

Currently, pollinators are facing increased vulnerability to infectious diseases and other negative environmental stressors, such as harmful pesticides [8]. A reported decline in the abundance and diversity of beneficial insect pollinators can be caused by virus infections. The wild bumblebee population is also under the threat of viral infections [9]. Viruses are spilling over from managed and imported honeybees to free-living insect pollinators, including bumblebees [10,11]. Consequently, there is the possibility of disease occurrence after direct transmission through shared contaminated floral resources or facilitated by changes in host immune status and susceptibility [1]. Moreover, recently, genetically identical strains of deformed wing virus (DWV) were detected in honeybee colonies (*Apis mellifera*) and in *Varroa destructor*, their obligate parasitic mite, making it a vector of this virus disease [10,12].

The importation and deployment of managed honeybee and bumblebee colonies may be a source of pathogen introductions in new geographical areas or alterations in the dynamics of native parasites and causative agents of secondary diseases, e.g., viruses, that ultimately increase disease prevalence in wild bees [1]. Insect pollinator decline has become a worldwide issue [4,5], causing increased concerns over effects on global food production [5], the stability of pollination services [13], and the disruption of the plant–pollinator link [14].

It is known that RNA honeybee viruses, due to their short generation of life and high mutation rates, have already crossed species barriers and have successfully infected a wide range of new insect hosts, such as free-living wild bees—solitary bees and bumblebees, wasps, hoverflies, and ants [9,15].

In recent years, there have been several reports of bumblebees infected with viral honeybee pathogens: DWV, black queen cell virus (BQCV), Israeli acute paralysis virus (IAPV), chronic bee paralysis virus (CBPV), Kashmir bee virus (KBV), and Sacbrood bee virus (SBV) [10,15,16]. In Croatia, the prevalence and regional distribution patterns of seven different honeybee viruses was studied, and simultaneous infection of adult honeybee samples with two to four different viruses was identified [17,18].

The aim of this research was to determine the presence and quantification of different honeybee viruses (ABPV, CBPV, BQCV, and DWV) in wild bumblebee samples originating from 27 geographically different locations. This is the first record of molecular viral examinations, as well as important new phylogenetic comparison information for endemic honeybee virus strains circulating in bumblebees in the territory of Croatia.

## 2. Results

**Figure 1:**
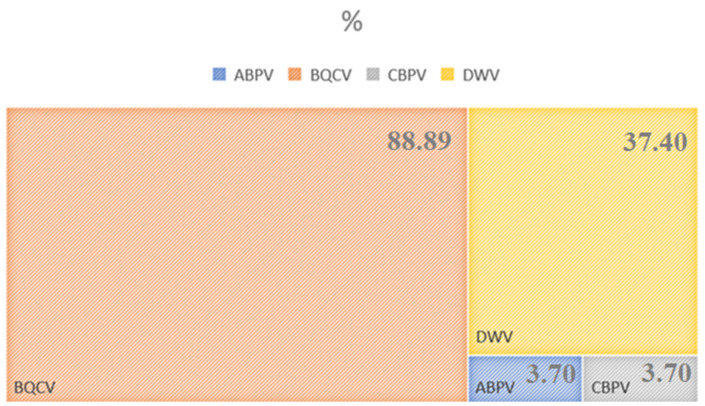
The determined prevalence (%) of the 27 tested bumblebee samples for the presence of four different honeybee viruses (ABPV, BQCV, CBPV, and DWV).

Collected samples of bumblebees, originating from different locations in Croatia, were found positive with prevalence for ABPV (3.70%), BQCV (88.89%), CBPV (3.70%), and DWV (37.40%) (Figure 1).

The obtained Ct value for one ABPV-positive sample was 35.04, corresponding to 9.082 × 10^2^ copy number/5 µL. Altogether, BQCV was detected in 24 out of 27 samples, with Ct values from the lowest 17.24 to the highest 29.71 (corresponding to copy number/5 µL from 1.251 × 10^4^ to 1.224 × 10^8^). One bumblebee sample was detected as CBPV-positive with Ct value 41.00. The detected Ct values for ten DWV-positive samples varied from 27.38 to 41.84 with copy number/5 µL from 6.33 to 6.392 × 10^4^ (Table 1).

**Figure 2:**
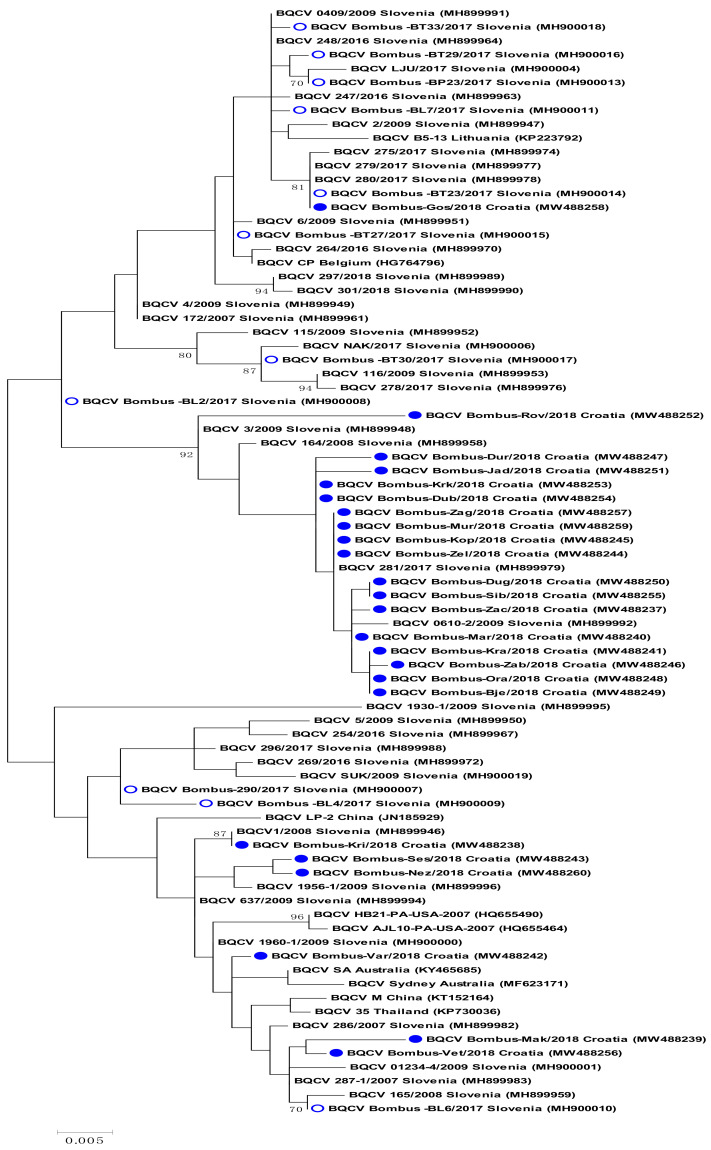
The BQCV tree was constructed from 653 nt long sequences of capsid protein gene (between nt positions 7774 and 8426; numbering according to BQCV isolate Sydney, MF623171) for 24 Croatian and 56 DWV isolates from GenBank. BQCV bumblebee samples from Croatia (●) and BQCV bumblebee positive samples from Genbank (O) are marked on the phylogenetic tree. The maximum likelihood phylogenetic tree with the Tamura 3-parameter substitution model with Gamma distribution. Statistical support for the tree was evaluated by bootstrapping, based on 1000 repetitions. Bootstrap values lower than 70 are not shown.

For 24 BQCV-positive bumblebee samples, the 653 nucleotide long sequences were successfully determined and compared with those available in GenBank. High genetic diversity among 24 Croatian sequenced BQCV-positive samples in bumblebee were identified, with 95.7 to 100% nucleotide identity to each other. The majority of the identified BQCV strains were closely related with BQCV stains from Slovenia. Strain BQCV Bombus-Gos/2018 (MW488258) has 100% nucleotide identity with bumblebee strain BQCV Bombus BT23/2017 (MH900014) and honeybee isolate 279/2017 (MH899977), both detected in the neighboring country of Slovenia. A group of 17 closely related BQCV-positive samples, located on the same branch of phylogenetic tree, were collected throughout the territory of Croatia, and have from 98.47 to 100% nucleotide identity with isolate 281/2017 (MH899979), detected in Slovenia. The BQCV strain Bombus-Var/2018 (MW488242) has 99.54% nucleotide identities with isolate BQCV 637/2009 (MH899994) detected in Slovenia and 98.82% nucleotide identity with strain Sydney ((MF623171) detected in Australia. Strain Bombus-Vet/2018 (MW488256) has 99.69% nucleotide identity with strain 287-1/2007 (MH899983), while with the strain Bombus-Mak/2018 (MW488239) has 99.08% identity. Detected strain Bombus-Kri/2018 (MW488238) collected in Križevci showed 100% nucleotide identity with Slovenian honeybee strain 1/2008 (MH899946). Strain Bombus-Ses/2018 (MW488243) and Bombus-Nez/2018 (MW488260) have 99.39% nucleotide identities with isolate 1956/2009 (MH899996) identified in Slovenia in 2009 in clinically affected honeybee samples (Figure 2).

**Figure 3:**
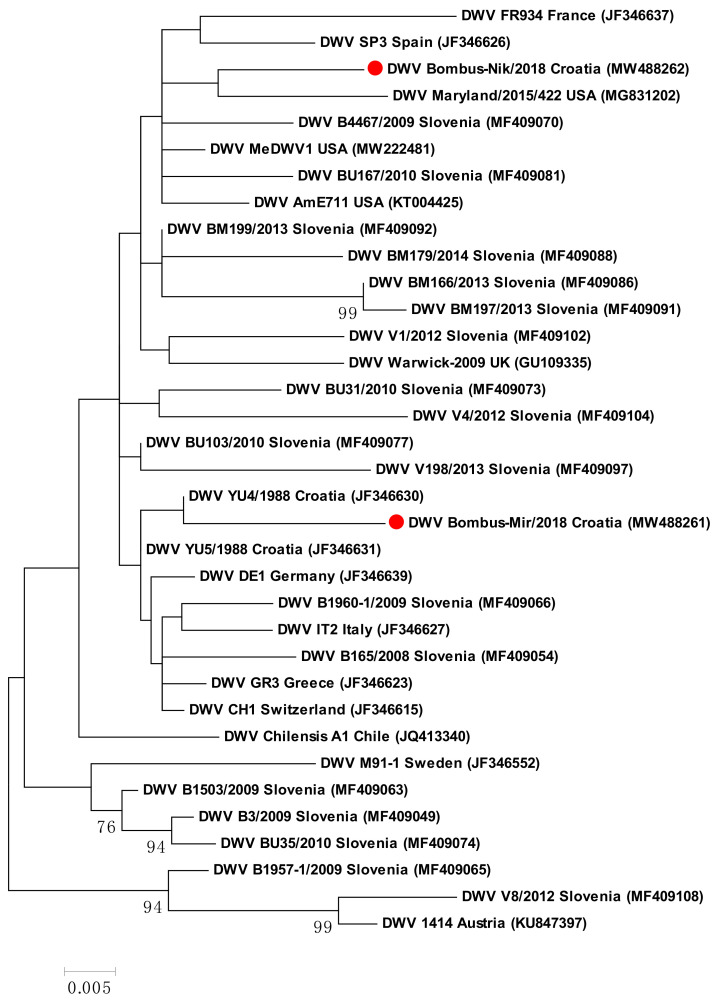
The DWV tree was constructed for two Croatian and 33 DWV isolates from GenBank from 471 nt long sequences of L protein gene (between nt positions 1357 and 1827; numbering according to isolate Austria 1414, KU847397). The two DWV bumblebee samples from Croatia determined in this study are marked (●) on the phylogenetic tree. The maximum likelihood phylogenetic tree with the Tamura 3-parameter substitution model with Gamma distribution. Statistical support for the tree was evaluated by bootstrapping, based on 1000 repetitions. Bootstrap values lower than 70 are not shown.

For 2 samples out of 10 DWV-positive bumblebee samples, the 471 nucleotide long sequences were successfully determined and compared with those available in GenBank. A DWV-positive sample Bombus-Mir/2018 (MW488261) was collected in 2018 in Zagreb and has 98.09% nucleotide identity with the closely related strains YU4 (JF346630) and YU5 (JF346631) in GenBank, collected in 1988 near Turopoljski Lug, which is located about 50 km from Zagreb city. The second DWV-positive sample Bombus-Nik/2018 (MW488262) was collected near Šibenik and has 98.09% nucleotide identity with strain SLO/BM199/2013, detected in *A. mellifera* in 2013 in Slovenia, while with DWV strain MeDWV1 (MW222481) and DWV strain Maryland/2015/422 (MG831202), both from the USA, it shares from 97.45 to 97.66% nucleotide identity (Figure 3).

Genetically closely related strains, previously detected in the same geographic region among honeybees, were also detected in randomly selected bumblebee samples in Croatia. Twenty-six determined sequences from this study were deposited in GenBank with accession numbers MW488237-MW488262.

## 3. Discussion

This study is the first attempt to constitute an epidemiological baseline regarding the geographical distribution patterns and prevalence of four honeybee viruses in wild bumblebee samples across the territory of Croatia. Results showed that a higher prevalence of BQCV and DWV was determined in tested wild bumblebee samples collected in the continental part of country where the honeybee colonies’ density is higher, in comparison with locations at the Adriatic coast and on islands. In particular, high BQCV prevalence was determined at all locations, except in samples originating from the Dalmatian islands of Ugljan and Pašman. Similar results were presented by Alger et al. (2019) and Toplak et al. (2020), which provided support to the observations that honeybee viruses are probably spilling over from managed honeybee colonies to wild bumblebees through visits to the same floral sources [10,11].

Since the current study does not include bumblebee species differentiation, we are not able to discuss possible differences in the species-specific vulnerability of bumblebees to tested honeybee viruses. However, according to Toplak et al. (2020) the different species of bumblebees tested in two consecutive years showed high variability in prevalence for different viruses and species. In addition, their results showed high variability for different species of bumblebees in a short period in the same geographic area [11]. Nevertheless, in our study, the important first data were obtained regarding the prevalence of four honeybee viruses on bumblebees from our region.

This research confirmed that genetically identical or closely related honeybee strains of BQCV and DWV were also identified among tested bumblebees, collected from the same location (geographic area). The high genetic identity with previously determined Slovenian BQCV and DWV strains is not surprising because of the neighboring area and historically long tradition of honeybee pasturing during spring and summer time. Furthermore, those two countries have a good beekeeper connection via trade. The identification of a group of 17 genetically closely related BQCV-positive samples, which were collected throughout territory of Croatia, is probably the result of recent years’ transmission events. The opposite observation with the identification of relatively high diversity of BQCV strains among bumblebees is the result of the persistence of this virus for a long period in the population and was like that observed in a previous study in Slovenia [10]. Although honeybee positive samples from Croatia were not included in this study, the genetically very similar strains of BQCV and DWV than identified in bumblebees could be expected in honeybees from the same territory. This was confirmed with the identification of DWV-positive sample Bombus-Mir/2018 (MW488261), which has 98.09% nucleotide identity with the two closely related DWV strains YU4 (JF346630) and YU5 (JF346631), collected more than 30 years ago in Croatia, both DWV-positive samples were collected in *A. mellifera*, from a location near Zagreb city [19].

Interestingly, ABPV and CBPV were detected only in one sample from one location each, with a very low viral load copy number. Due to low positive samples of both ABPV and CBPV, the sequencing and phylogenetic analyses were not possible in this study. In contrast, ten examined bumblebees’ samples were DWV-positive (37.40%), which is significantly higher infection prevalence than results presented in previously published studies, where the range was 2.70 to 11% positive bumblebees’ samples [9,10,11,15]. However, general observation for both BQCV- and DWV-positive samples showed that low copy numbers were identified in each pool of five bumblebees’ positive sample, suggesting that these viruses are present in bumblebees, but they may have limited impact on bumblebee pathology. Tehel et al. (2020) reported higher viral titers of BQCV, DWV genotype A, and DWV genotype B in bumblebees after experimental inoculation of a pathogen by injection in comparison with oral inoculation [20]. Namely, among more than 30 honeybee-infecting known viruses [21,22], three are characterized by specific clinical symptoms: CBPV, DWV, and SBV [23]. BQCV and ABPV with its belonging complex can show alterations in the morphology of developmental stages of honeybees and adults’ behavior. Furthermore, some can be present as inapparent or subclinical infections [24].

It is not yet completely ascertained if field viruses’ strains are able to cause clinical manifestation in bumblebees. It is also not clear which environmental stressors are promoting factors for converting an asymptomatic infection into symptomatic and overt. In this study, all collected bumblebees were without visible morphological or behavioral changes, so they were considered clinically healthy. Although the virus presence could also be detected in individual bumblebee tissues or organs, this approach was not applied in our study and was assessed firstly to define the prevalence and diversity of four tested viruses. However, for further research it would be good to use individual bumblebee tissues for the estimation of individual virus tropism for specific tissue and for evaluating the possible effects that those viruses may have on their bumblebee hosts. In addition, according to Manley et al. (2019), honeybee parasitic mite *V. destructor* drives DWV prevalence and titer in honeybees and wild bumblebees [25]. Similarly, experimental injections of DWV under laboratory conditions or natural direct inoculation through *V. destructor* host feeding into insect body haemocoel causes an increase in prevalence and virulence in honeybees [26,27].

In our study, BQCV-positive samples were determined in very high prevalence (24 positive samples/27 total number of samples), which is contrary to the published findings of Dolezal et al. (2016), where same virus was detected extremely rarely in wild bees [28]. However, a recent publication from Slovenia, with the identification of genetically identical strains of ABPV, BQCV, SBV, and Lake Sinai virus (LSV) supports the observation of our study, that identical strains are present in honeybees and bumblebees [10]. Previously published data from Croatian honeybee samples originating from 82 apiaries located in 20 different districts showed a wide spread of honeybee viruses, with 9.75% of CBPV-positive samples, while ABPV, BQCV, and DWV were found in 10.97%, 40.24%, and 95.12% of tested apiaries, respectively [17]. Simultaneous infections with a maximum of two different viruses were detected in 10 (37.03%) of 27 bumblebee samples, and this was lower than the previously observed 64.6% of multiple infections among tested honeybees in Croatia [18].

## 4. Materials and Methods

### 4.1. Field Sampling

**Figure 4:**
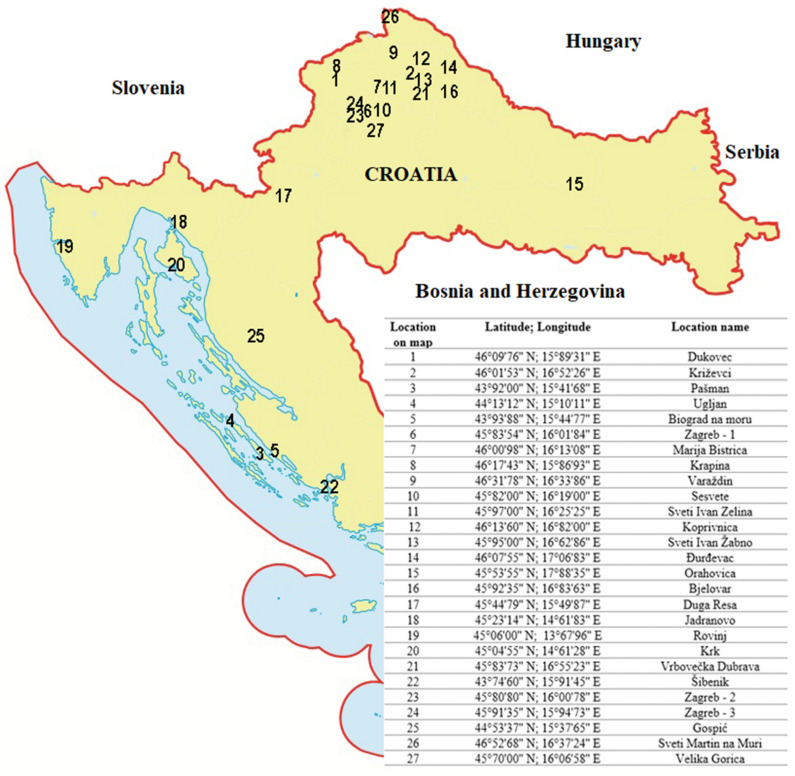
Sampling location sites (sample number) where a total of 27 samples of healthy bumblebees was collected during July and August 2018. The sample numbers represent different locations on the map of Croatian territory corresponding to the names of those locations, as indicated in accompanying table.

To determine the presence and the prevalence of four different honeybee viruses (ABPV, BQCV, CBPV, and DWV) in wild bumblebees (*Bombus terrestris*, *Bombus lapidarius*, *Bombus pascuorum*), 27 randomly selected bumblebee samples were collected from a total of 27 sampling locations from the territory of Croatia (Figure 4). Sampling was conducted during July and August 2018. At each site, five clinically healthy bumblebees were taken from flowers, representing one pool sample from each location. Each sample was marked with the number of the sampling location. Collected samples of bumblebees were stored under –70 °C until the start of molecular analyses.

### 4.2. Molecular Analyses in Laboratory Conditions

For RNA extraction purposes, each pooled sample consisting of five bumblebees’ specimens was placed into Ultra-Turrax DT-20 tubes (IKA, Königswinter, Germany) with five mL of RPMI 1640 medium (Gibco, Paisley, UK) and incubated at room temperature for 30 min. Then, prepared samples were homogenized and centrifuged for 15 min at 2500× *g*. Two milliliters of supernatant were stored from each sample as a suspension for further extraction.

Primers, TaqMan probes, and quantification standards for ABPV [29], BQCV [30], CBPV [31], and DWV [32] were set with reagents according to previously published protocol.

Briefly, the total RNA was extracted from 140 µL of suspension from each sample by the QIAamp viral RNA mini-kit (Qiagen, Hilden, Germany) and recovered from a spin column in 60 µL of elution buffer. Reverse transcription with RT-qPCR assay was made in a single step using QuantiNova Pathogen + IC Kit (Qiagen, Hilden, Germany). The RT-qPCR mix consisted of 5 µL QuantiNova Master Mix, 2 µL 10× Internal (inhibition) Control (IC) Probe Assay, 1 µL IC (1:100), 4.5 µL deionized water, 1 µL forward primer (200 nM), 1 µL reverse primer (200 nM), and 0.5 µL probe (100 nM) and 5 µL of extracted RNA with a total of 20 µL final volume. Thermal cycling was performed on Mx3005P thermocycler (Stratagene, La Jolla, CA, USA) with the following conditions: 20 min 50 °C, 2 min 95 °C, followed by 45 cycles of 15 s 95 °C, 30 s 60 °C, and 30 s 60 °C. In each run, the positive control was included, prepared as a mixed suspension of previously determined positive field samples of four different viruses (ABPV, BQCV, CBPV, and DWV). The negative control was prepared and used in the same way as the positive, while each negative control consisted only of 160 µL of RPMI 1640 medium (Gibco, Paisely, UK) in aliquot.

### 4.3. Data Processing, Statistical Analysis, and Reporting

The known copy number of the standard for each virus, with 10-fold dilutions from 10^−3^ to 10^−7^, were prepared and added in each RT-qPCR run. The exact number of RNA viral molecules in individual sample was calculated for positive samples from the standard curve for each of the four honeybee viruses.

The results for each sample were analyzed using MxPro-Mx3005P v4.10 software (Stratagene, La Jolla, CA, USA) and the exact copy number was determined from the standard curve. Results were expressed as number of detected viral copies in 5 µL of extracted RNA.

For sequencing purpose, the BQCV- and DWV-positive samples were amplified by using a specific method of reverse transcription and polymerase chain reaction (RT-PCR), as previously described [33]. Results were evaluated based on the size of RT-PCR products in the agarose gel as positive in the case of the expected product size: for BQCV, 770 nt, and for DWV, 504 nt [33]. In the case of a positive result, the selected RT-PCR products of a single virus were directly sequenced with the Sanger sequencing protocol, using the same primers as used for specific RT-PCR as described previously [10]. Individual sequences were analyzed using the DNASTAR 5.05 (Lasergen, WI, USA) program, and 26 positive samples of two viruses (BQCV *n* = 24 and DWV *n* = 2) were detected in bumblebees together with closely related sequences from GenBank and interpreted according to the results of the nucleotide sequence matching between honeybee and bumblebee samples. Multiple alignments were created using the program MEGA 6.06. Genetic distances were calculated from the alignment based on the Tamura three-parameter model, and phylogenetic trees were generated using the maximum likelihood (ML) statistical method implemented with the Tamura 3-parameter model with Gamma distribution [34]. The test of phylogeny was performed through 1000 bootstrap replicates. Only bootstrap values higher than 70% were presented on phylogenetic trees. The comparative analyses of collected samples at 27 locations and comparison with previously detected viruses in honeybees and with the most closely related sequences available in GenBank were performed.

## 5. Conclusions

Results of this research confirm that several honeybee viruses (ABPV, CBPV, BQCV, DWV) were found in wild bumblebees in Croatia. It can also be concluded that the presence of the examined viruses was higher in continental parts of the country compared with the Adriatic coast and islands. However, because the virus’s presence was not studied in internal bumblebee tissues of separate bumblebee species, in further studies, samples of different bumblebee species will be tested individually to define species-specific prevalence and to evaluate the possible impact that those viruses may have on their bumblebee hosts. Moreover, due to the neighborhood and a good beekeeper connection via trade, virus genetic matches were determined in Croatia and Slovenia.

## Figures and Tables

**Table 1:** The determined copy number in 27 tested bumblebee samples by real-time RT-PCR assay for the detection of ABPV, BQCV, CBPV, and DWV, collected from 27 different geographical locations in Croatia.

Sample Number	Name of Sample	ABPV Copy Number/5 µL	BQCV Copy Number/5 µL	CBPV Copy Number/5 µL	DWV Copy Number/5 µL	Number of Viruses
1	Bombus-Zac/2018	9.082 × 10^2^	1.251 × 10^4^	0	0	2
2	Bombus-Kri/2018	0	4.415 × 10^6^	0	0	1
3	Bombus-Pas/2018	0	0	0	0	0
4	Bombus-Uglj/2018	0	0	0	1.739 × 10^2^	1
5	Bombus-Bnm/18	0	0	0	1.257 × 10^4^	1
6	Bombus-Mak/2018	0	9.160 × 10^7^	0	1.260 × 10^4^	2
7	Bombus-Mar/2018	0	2.198 × 10^6^	0	0	1
8	Bombus-Kra/2018	0	4.212 × 10^6^	0	0	1
9	Bombus-Var/2018	0	3.511 × 10^6^	0	0	1
10	Bombus-Ses/2018	0	18.09 × 10^7^	0	6.234 × 10	2
11	Bombus-Zel/2018	0	25.83 × 10^5^	0	0	1
12	Bombus-Kop/2018	0	2.257 × 10^5^	0	0	1
13	Bombus-Zab/2018	0	1.678 × 10^6^	0	0	1
14	Bombus-Dur/2018	0	2.777 × 10^6^	0	0	1
15	Bombus-Ora/2018	0	4.400 × 10^5^	0	0	1
16	Bombus-Bje/2018	0	1.042 × 10^6^	0	0	1
17	Bombus-Dug/2018	0	2.131 × 10^5^	0	0	1
18	Bombus-Jad/2018	0	3.200 × 10^7^	0	3.351 × 10	2
19	Bombus-Rov/2018	0	1.588 × 10^5^	0	0	1
20	Bombus-Krk/2018	0	8.975 × 10^3^	0	3.08	2
21	Bombus-Dub/2018	0	1.656 × 10^5^	6.33	0	2
22	Bombus-Sib/2018	0	1.110 × 10^6^	0	6.392 × 10^4^	2
23	Bombus-Vet/2018	0	7.72 × 10^5^	0	1.285 × 10^2^	2
24	Bombus-Zag/2018	0	1.309 × 10^5^	0	0	1
25	Bombus-Gos/2018	0	1.348 × 10^5^	0	3.741 × 10^2^	2
26	Bombus-Mur/2018	0	1.360 × 10^5^	0	0	1
27	Bombus-Nez/2018	0	1.224 × 10^8^	0	1.140 × 10^3^	2

## Data Availability

Data are presented in the manuscript. Twenty-six determined sequences from this study were deposited in GenBank with accession numbers MW488237-MW488262.
